# Effect of bovine adenovirus 3 on mitochondria

**DOI:** 10.1186/1297-9716-45-45

**Published:** 2014-04-16

**Authors:** Sanjeev K Anand, Jaswant Singh, Amit Gaba, Suresh K Tikoo

**Affiliations:** 1 Vaccine and Infectious Disease Organization –International Vaccine Center (VIDO- InterVac), University of Saskatchewan, Saskatoon, SK S7N 5E3, Canada; 2 Veterinary Microbiology, Western College of Veterinary Medicine, University of Saskatchewan, Saskatoon, SK S7N 5B4, Canada; 3 Veterinary Biomedical Sciences, Western College of Veterinary Medicine, University of Saskatchewan, Saskatoon, SK S7N 5B4, Canada; 4 Vaccinology & Immunotherapeutics program, School of Public Health, University of Saskatchewan, Saskatoon, SK S7N 5E5, Canada

## Abstract

Viruses alter the structure and the function of mitochondria for survival. Electron microscopy analysis of the cells infected with bovine adenovirus 3 revealed extensive damage to the inner mitochondrial membrane characterized by dissolution of the cristae and amorphous appearance of mitochondrial matrix with little or no damage to the outer mitochondrial membrane. There were fewer cristae with altered morphology. Potential patches of protein synthesis machinary around mitochondria could be observed at 12 hours post infection (hpi). At 24 hpi, the multi vascular bodies were evident throughout the infected cell. ATP production, mitochondrial Ca^2+^ and mitochondrial membrane potential (MMP) peaked at 18 hpi but decreased significantly at 24 hpi. This decrease coincided with the increased production of superoxide (SO) and reactive oxygen species (ROS), at 24 hpi indicating acute oxidative stress in the cells and suggesting a complete failure of the cellular homeostatic machinary. The results reveal an intericate relationship between Ca^2+^ homeostasis, the ATP generation ability of cells, SO and ROS production, and regulation of MMP following infection by bovine adenovirus 3.

## Introduction

Mitochondria are cytoplasmic organelles found in the eukaryotic cells. Their number and size varies from depending upon the function and the metabolic state of the cell. Although mitochondria have their own genome and transcription-translation machinery, they also depend on nuclear encoded gene products, which are indispensable for their normal function [1]. Besides acting as power houses of the cell, they are important for regulation of calcium and cellular metabolism [2] and play a central role in apoptosis [3]. Mitochondria have also been implicated in aging [3], development [4], antiviral responses [4] signal transduction [5] and diseases [6]. In short, they control the main processes critical for the survival of the cell. Thus, they have developed a very intimate and complex relationship with the cell, some of which is still elusive.

A number of viruses can affect the structure and the function of the mitochondria including oxidative stress, loss of mitochondrial membrane potential (MMP), and even depletion of the mitochondrial (mt) DNA [7].

Though adenovirus replicates in the nucleus of the cell, the possibility of its dependence on the mitochondria can’t be ruled out. However, little is known about the role of mitochondria in adenovirus infections. Earlier reports reported the increase in the cellular respiration during release of adenovirus from the infected cells suggesting the role of mitochondria [8]. Some human adenovirus early proteins localize to the mitochondria and prevent apoptosis [9]. Adenovirus death protein (ADP) encoded by E3 region of human adenoviruses has been implicated in the release of virus progeny from the infected cell after successful replication.

Bovine adenovirus 3 (BAdV-3), a non-enveloped icosahedral particle of 75-nm diameter replicates in the respiratory tract of cattle with mild or no clinical symptoms [10]. The complete DNA sequence and the transcription map of BAdV-3 genome have been reported [11]. As little is known about BAdV-3-host interaction, the present study aims to identify the effect of BAdV-3 replication on the mitochondria of infected cells.

## Materials and methods

### Cell culture

Madin-Darby bovine kidney (MDBK) cells were grown in minimal essential medium (MEM; Invitrogen, Burlington, ON, Canada) supplemented with 10% heat-inactivated fetal bovine serum. Wild-type BAdV-3 (WBR-1 strain) was propagated in MDBK cells in MEM supplemented with 2% FBS [12].

### Antibodies

Production of polyclonal antibody specific to 19 kDa E1B protein of BAdV-3 is described elsewhere [13]. Antibody specific to 42 kDa β-actin protein was purchased from Sigma, (Mississauga, ON, Canada) (Cat # A5441). Alkaline phosphatase (AP)-conjugated goat anti-rabbit IgG (Cat # 111-055-003) or AP-conjugated goat anti-mouse antibody (Cat # 115-055-003) was purchased from Jackson ImmunoResearch, Philadelphia, USA.

### Western blot analysis

Proteins from BAdV-3 infected cells were analyzed by Western blot as described earlier [12].

### Transmission electron microscopy

The electron microscopy was performed as described earlier [12].

### Analysis of mitochondrial processes

MDBK cells were grown in 96-well plates and infected with BAdV-3 at an MOI of 5. At 0, 6, 12, 18 and 24 hpi, the infected or the uninfected cells were analyzed for cellular ATP production, levels of mitochondrial and cytosolic Ca^2+^, alteration in mitochondrial membrane potential, and production of mitochondrial reactive oxygen species (ROS) and Super oxide (S0) as described earlier [14].

### Statistical analysis

Fluorescence data measured from “30000-40000” cells on an average and plotted as an average of 6 independent experiments for each function was collected and analyzed as described [14,15].

### Computer programs

All pictures have been generated using power point program included in Microsoft Office: Mac2011.

## Results

### BAdV-3 damages mitochondrial architecture

**Figure 1:**
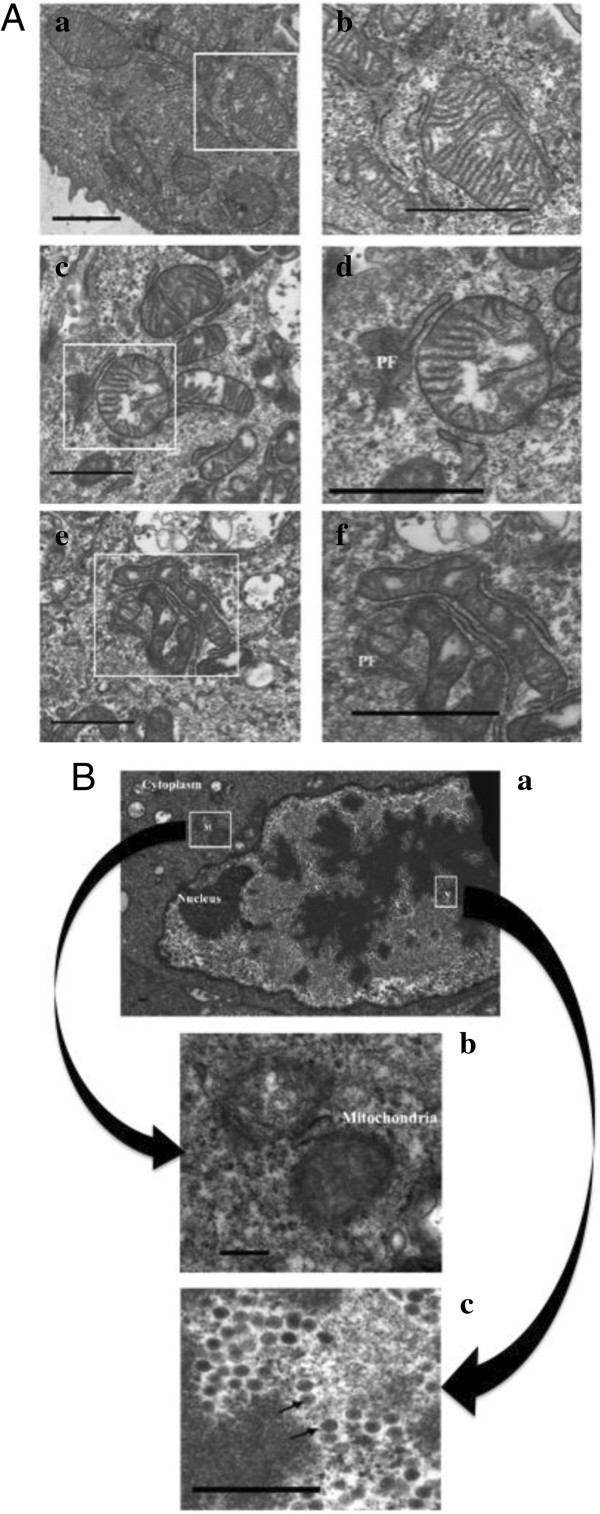
**Electron microscopy of BAdV-3 infected cells. (A)** MDBK cells mock infected (panel a, b) or infected with wild-type BAdV-3 (panels c, d, e, f) at a MOI of 5 were analyzed after 6 (panel c, d) and 12 (panel e, f) hpi. Figures in the left panels (panels a, c, e) show the cytoplasm in the vicinity of the mitochondria. Area covered by white rectangles is enlarged and shown in the panels (panel b, d, f) on the right. Protein factories (PF) in the vicinity of the damaged mitochondria at 6 (panel d) and 12 (panel f) hpi. Bar = 0.25 μ. **(B)** MDBK cells infected with wild-type BAdV-3 at an MOI of 5 were analyzed at 24 hpi. An infected cell showing the virus particles (V) in the nucleus and the damaged mitochondria (M) in the cytoplasm (panel a). The enlarged area indicated by a rectangle (M) in panel “a” showing the mitochondria with amorphous internal structure in the cytoplasm of the infected cell (panel b). The enlarged area indicated by a rectangle (V) in panel “a” showing virus particles (indicated by arrows) in the nucleus of infected cells (panel c). Bar = 0.25 μ.

To evaluate the effect of BAdV-3 on mitochondria during the course of infection, electron microscopy was used. As seen in Figure 1A, the uninfected cells showed normal lamellar bodies with rough endoplasmic reticulum (rER) and many polyribosomes. Majority of the mitochondria were located in the peri-nuclear or central 2/3^rd^ of the cytoplasm. Mitochondria were elongated or oval in shape containing typical cristae of protein producing cells (panel a, b). At 6 hpi (panel c, d), mitochondria still appeared to have normal morphology with intact outer and the inner membranes. Although there were increased cristae free zones in the mitochondria, the density of the cristae appeared to be normal.

At 12 hpi (panel e, f), the mitochondria of the infected cells appeared to be smaller in size compared to the mitochondria of the normal cells (panel a, b). Although morphology was intact, the cristae free zones increased in the virus infected cell mitochondria at 12 hpi compared to the infected cell mitochondria at 0 or 6 hpi. At 24 hpi (Figure 1B, panel a, b), the cells had longer microvilli on the surface and with fewer areas of a cell in contact with the other. The nuclear membrane was more indented with the appearance of nuclear inclusions. The progeny virus particles were observed inside the nucleus with the decrease in the number of nucleoli per cell. Moreover, the mitochondria appeared smaller and round/oval in shape. Interestingly, very few mitochondria were observed at this time and patches of the protein synthesis mahinary were not visible around the mitochondria.

### BAdV-3 regulates ATP production in infected cells

**Figure 2:**
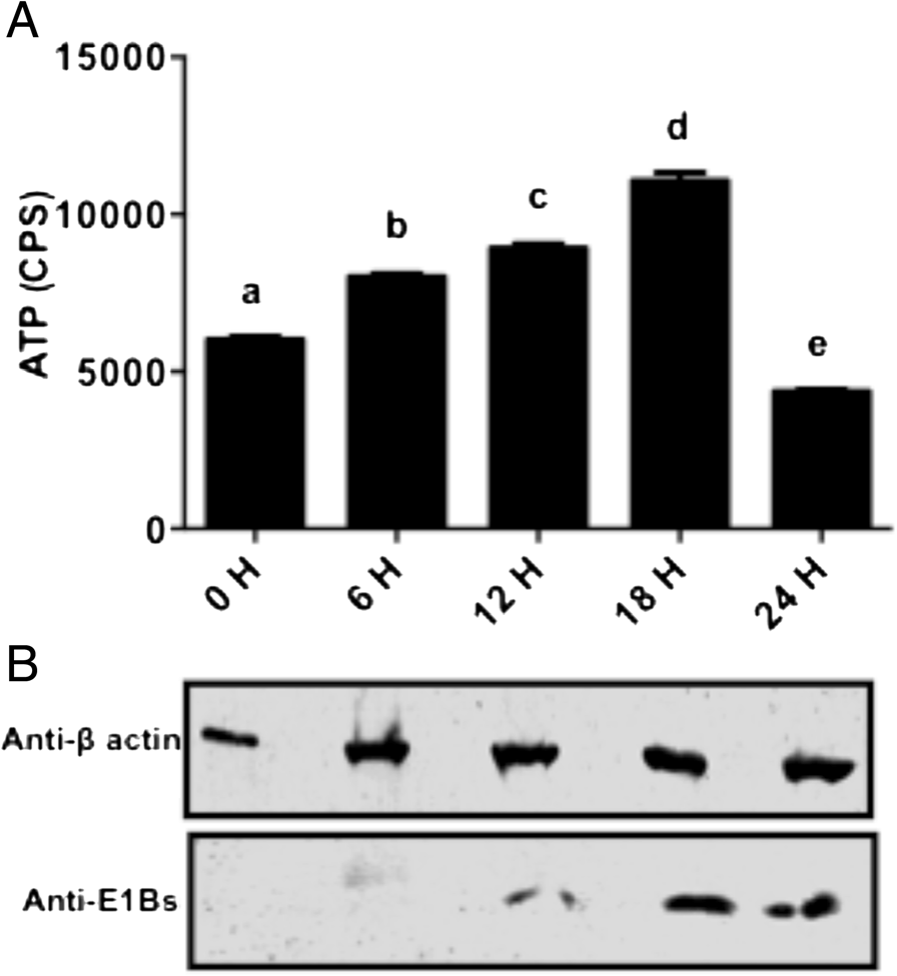
**ATP production in BAdV-3 infected cells. (A)** Data represents the mean of 2 independent experiments, each with 3 replicates. Means with the different letter are significantly different. * *P* < 0.0001 **(B)** Western blot analysis of BAdV-3 infected cells using anti-β-actin serum (Sigma, Mississauga, ON, Canada) or anti -E1aBs serum [31].

Since BAdV-3 affects the mitochondrial morphology, we determined if ATP production capacity of the cells is compromised during the course of infection. ATP is present in all metabolically active cells and thus, is a marker for the cell viability. Whenever the cell is under stress, ATP concentration changes rapidly and thus, monitoring ATP is a good indicator of cell health. As seen in Figure 2, steady increase in ATP production was observed at 6, 12 and 18 hpi. However, compared to 0, 6, 12 and 18 h, there was a significant (*P* < 0.0001) decrease in the ATP production at 24 hpi (Figure 2).

### BAdV-3 infection causes loss in mitochondrial membrane potential (MMP) in MDBK cells

**Figure 3:**
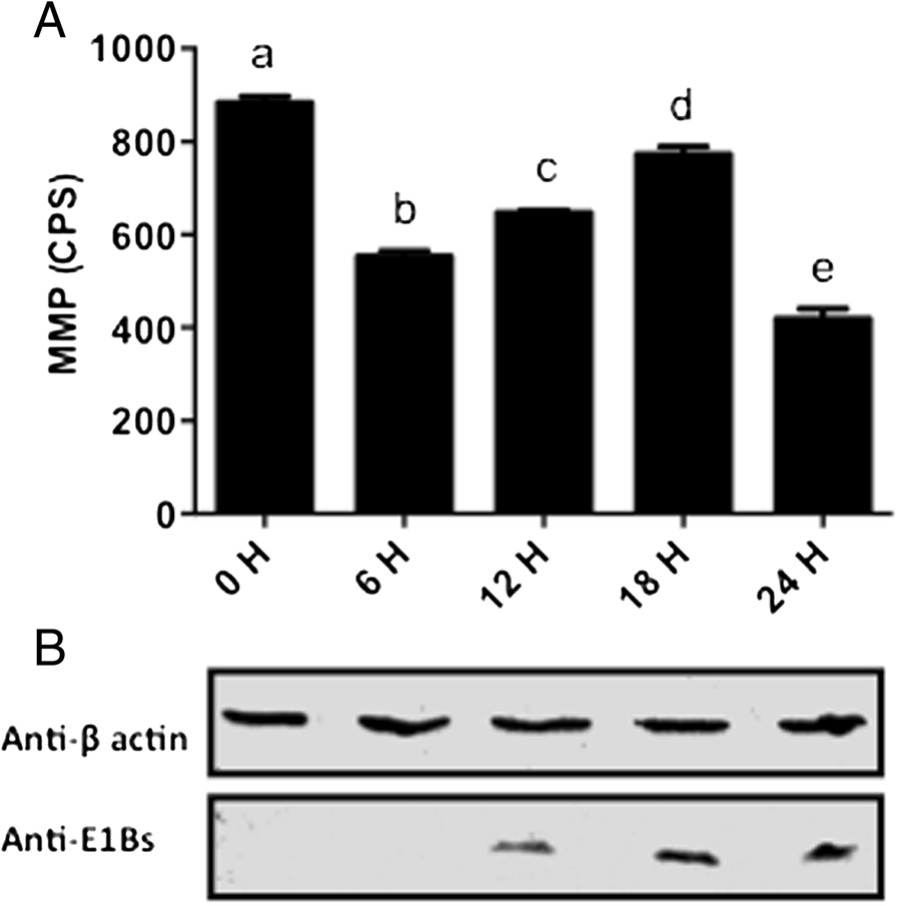
**Mitochondrial membrane potential in BAdV-3 infected cells. (A)** Data represents the mean of 2 independent experiments, each with 3 replicates. Means with the different letter are significantly different. * *P* < 0.0001. **(B)** Western blot analysis of BAdV-3 infected cells using anti-β-actin MAb (Sigma, Mississauga, ON, Canada) or anti -E1Bs serum [13].

As changes were observed in mitochondrial morphology and ATP production in BAdV-3 infected cells, we next determined if BAdV-3 alters the MMP in the virus-infected cells (Figure 3). TMRM is a monovalent cationic mitochondrial selective probe, which exhibits fluorescence when it accumulates in MMP dependent manner. Under the conditions of the mitochondrial depolarization, TMRM diffuses out and becomes more evenly distributed throughout the cytoplasm. When dispersed, the fluorescence drops significantly, which can be quantified. Quantification of the TMRM signals indicated that there was a significant decrease (at *P* < 0.0001) in the MMP in the cells from 0 to 6 hpi. In contrast, compared to 0 and 6 h there was a significant (*P* < 0.0001) increase in the MMP in the cells from 6 to 18 hpi. However, compared to 0, 6, 12 and 18 h, there was a significant (*P* < 0.0001) decrease in the MMP in the cells at 24 hpi.

### BAdV-3 infection causes decrease in mitochondrial Ca^2+^ levels in MDBK cells

**Figure 4:**
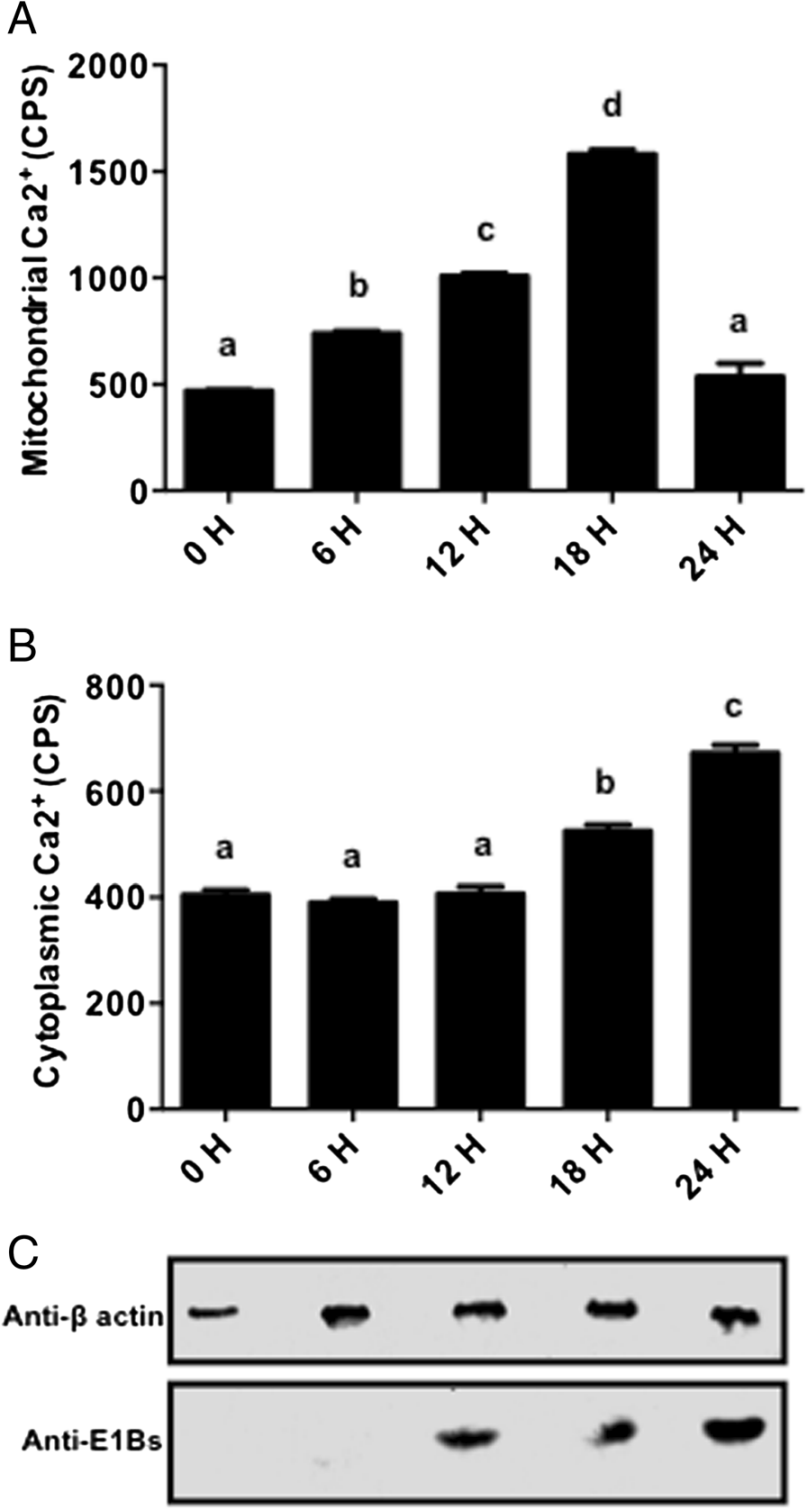
**Ca**^**2+ **^**in BAdV-3 infected cells. (A)** Mitochondrial Ca^2+^. Data represents the mean of 2 independent experiments, each with 3 replicates. Means with the different letter are significantly different. Means with the same letter are not significantly different. * *P* < 0.0001. **(B)** Cytosolic Ca^2+^. Data represents the mean of 2 independent experiments, each with 3 replicates. Means with the same letter are not significantly different. * *P* < 0.0001. **(C)** Western Blot analysis of BAdV-3 infected cells using anti-β-actin MAb (Sigma, Mississauga, ON, Canada) or anti -E1Bs serum [13].

Since Ca^2+^ buffering capacity of the mitochondria is a good indicator of mitochondrial health and survival in the cells, the mitochondrial Ca^2+^ levels were measured after incubating BAdV-3 infected cells with Rhod-2 AM, a high affinity mitochondrial Ca^2+^ indicator [16]. Rhod-2 AM binds specifically with the mitochondrial Ca^2+^ and fluorescence can be quantified using a multi label reader. As seen in Figure 4A, the mitochondrial Ca^2+^ levels were found to be significantly higher at 6, 12 and 18 h post BAdV-3 infection. However, compared to 0, 6, 12 and 18 h, there was a significant decrease in the mitochondrial Ca^2+^ levels from 18 hpi (Figure 4A). Similarly, cellular Ca^2+^ levels were measured by incubating BAdV-3 infected cells with Fluo-4 AM, a highly specific fluorescent dye for measuring the cytosolic Ca^2+^ levels in the cells [17]. Interestingly, there was no significant (*P* < 0.0001) change in the cytosolic Ca^2+^ levels of the infected cells from 0 to 12 hpi (Figure 4B). In contrast, compared to 0, 6 and 12 h, there was significant (*P* < 0.0001) increase in the cytosolic Ca^2+^ levels of the infected cells at 18 and 24 hpi (Figure 4B).

### BAdV-3 infection aggravates the ROS & superoxide production in MDBK cells

**Figure 5:**
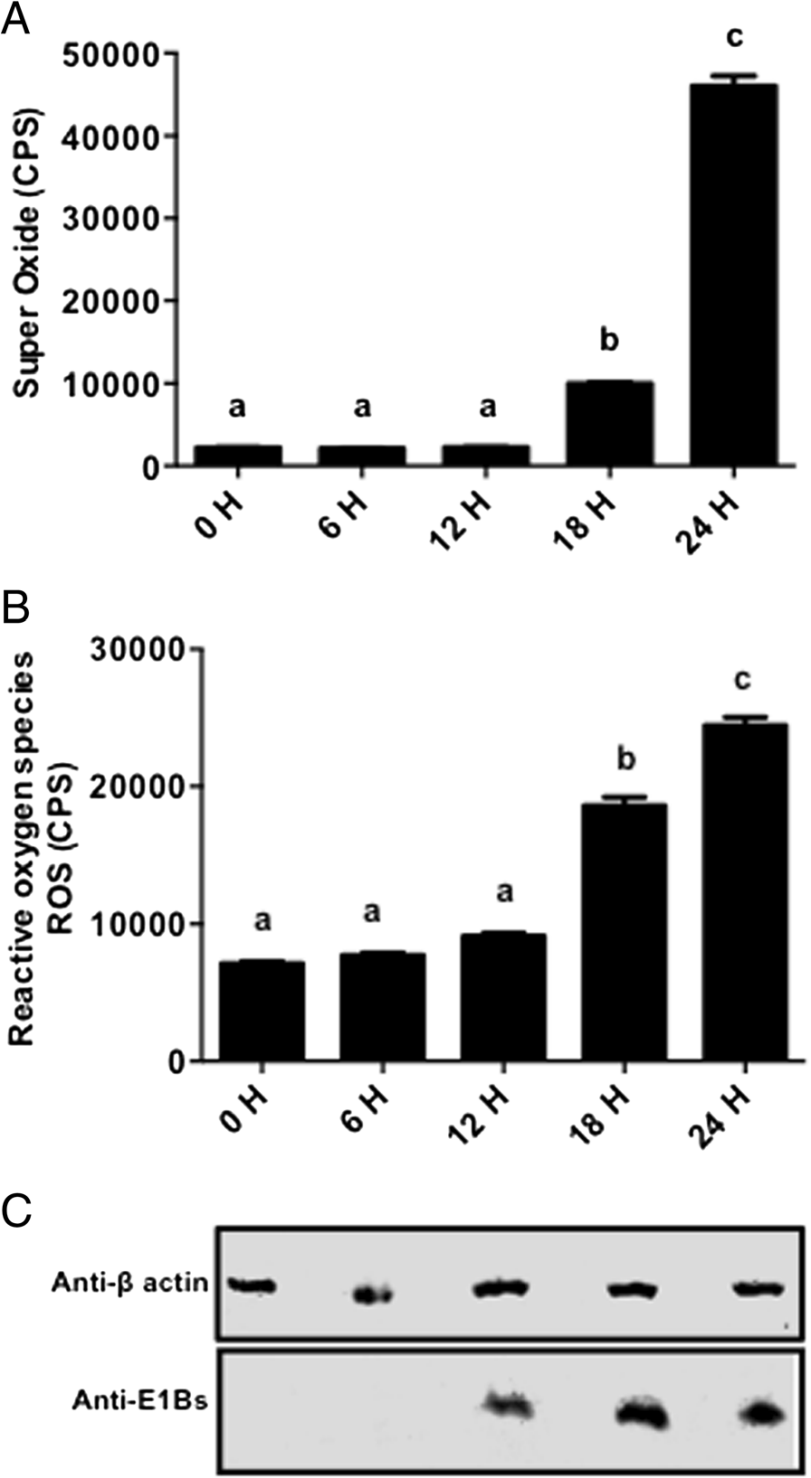
**Induction of Superoxide (SO) and reactive oxygen species (ROS) in BAdV-3 infected cells. (A)** SO production. Data represents the mean of 2 independent experiments, each with 3 replicates. Means with the same letter are not significantly different. * *P* < 0.0001. **(B)** ROS production. Data represents the mean of 2 independent experiments, each with 3 replicates. Means with the different letter are significantly different. Means with the same letter are not significantly different. * *P* < 0.0001. **(C)** Western blot of BAdV-3 infected cells using anti-β-actin MAb (Sigma, Mississauga, ON, Canada) or anti -E1Bs serum [13].

To assess the respiratory function of the cells infected with BAdV-3, both reactive oxygen species (ROS) and SO production was measured. MitoSOX™ Red reagent, a highly specific indicator of SO, is specifically targeted to the mitochondria and fluoresces when oxidized by SO but not by other ROS or reactive nitrogen species (RNS) generating systems [18]. Similarly, DCF-DA is sensitive to all the other ROS except SO [19]. As seen in Figure 5A, compared to 0, 6 and 12 h, the increased levels of SO were observed at 18 hpi, which were significantly higher at 24 hpi. Similarly, compared to 0, 6 and 12 h, the increased levels of ROS were observed at 18 h, which were significantly higher at 24 hpi (Figure 5B).

## Discussion

Mitochondria perform various functions that make them absolutely indispensable for the cell [3]. Besides acting as a powerhouse, they act as a common platform for the execution of a variety of cellular functions in the normal cells or in the cells under attack from the microorganisms including viruses [4]. A number of viruses affect the structure and the function of the mitochondria [7]. Here, we report the effects of BAdV-3 on the mitochondria during the normal course of infection of bovine cells.

Electron microscopy of BAdV-3 infected cells revealed extensive damage to the inner mitochondrial membrane characterized by the dissolution of cristae and amorphous appearance of the mitochondrial matrix while the outer mitochondrial membrane was observed to be intact. Various mitochondria specific lesions including degeneration of the mitochondria and dissolution of the mitochondrial cristae have been documented in Tobacco mosaic virus [20], Western Equine Encephalomyelitis (WEE) virus [21], vaccinia and fowl pox virus [22], and Echo virus type 9 [23] infected cells. Such changes in the mitochondria have been attributed to the dissipation of mitochondria membrane potential [24] due to opening of the membrane permeability transition pores [25]. Thus, invading viruses may be eliciting damage to the cristae by decreasing the synthesis or blocking the transport of the mitochondria specific proteins responsible for the maintenance of the inner mitochondrial membrane.

Different steps in viral replication including DNA packaging and capsid maturation require ATP [26]. Analysis of the ATP production during the course of BAdV-3 infection showed a steady increase in the ATP production till 18^th^ hpi, when the production of progeny virus particles is at its peak. As expected, the ATP levels decline after 18 hpi. This decline is in agreement with the culmination of the life cycle of BAdV-3. Variation in ATP production has also been associated with different stages of the viral life cycle indicating differential ATP requirements during the course of infection [27]. Increased level of ATP increases the viral replication including the release of vaccinia virus [28] and virus budding in influenza A virus [29] infected cells.

ATP is also required for the maintenance of most of the cellular and the mitochondrial functions [30]. Therefore, any change in cellular ATP production capacity will have direct impact on the membrane gradients inside the cell including the mitochondrial membrane potential (MMP). Our studies indicate that BAdV-3 modulates the MMP significantly at different times post infection. Initial transient decrease in MMP from 0–6 h followed by increase from 6–18 h may be due the expression of early adenoviral proteins. The role of adenovirus early proteins including E1A [31], E1B 19 K [32] and E4 orf4 [33] in the regulation of the longevity of the cell has been reported. A number of viruses including myxoma virus [34], hepatitis C virus (HCV) [35] and human immunedeficiency virus 1 (HIV-1) [36] modulate the MMP for their benefit by altering the activity of one or more components viz., the permeability transition pore (PTP) [37], the voltage dependent anionic channels (VDACs) [38] and the membranes. As expected, at 24 hpi, the MMP showed significant decrease, which coincides with the observed damage to the mitochondria and decreased ATP levels. Thus, increased ATP levels and prevention of the loss of MMP results in the prevention of the cell death, which is beneficial for the replication of BAdV-3. It is tempting to speculate that one or more BAdV-3 protein (s) may be involved in interactions with the mitochondria to help in increasing the ATP levels and the MMP. Recently, we reported that BAdV-3 pVII protein increases the ATP levels and maintain the MMP in transfected cells [14].

Ca^2+^ is one of the most abundant and most universal signal carriers which acts as a second messenger to regulate many cellular processes [39] including the ATP synthesis [40] and maintenance of the MMP [41]. The ATP levels [40] and the MMP regulate the Ca^2+^ homeostasis in the cells [42] and *vice versa* indicating a complex relationship between them. Our study didn’t show a significant shift in cytosolic Ca^2+^ levels. In contrast, the mitochondrial Ca^2+^ was observed to have peak retention at 18 hpi, similar to what we observed for the ATP and the MMP. We believe that BAdV-3 causes the retention of Ca^2+^ in the mitochondria, which leads to increase in the ATP synthesis, thus helping in the maintenance of the MMP. In addition to mitochondria, ER acts as a major source of Ca^2+^ in the cell. It is possible that whatever is the Ca^2+^, mitochondria uptake and retain during the process, ER releases the equivalent to make it up so that the cytosolic Ca^2+^ concentration remains the same. Thus, increase in the mitochondrial Ca^2+^ leads to increase in the ATP, the MMP and decrease in the ROS, which in turn may alter the apoptotic signalling [43]. Alterations in the mitochondrial Ca^2+^ levels [44] have been reported during HCV or herpes simplex virus 1 (HSV-1) [45] infection.

A variety of cellular defence mechanisms and enzymes including superoxide dismutase, catalases, lacto peroxidases, glutathione peroxidases and peroxiredoxins maintain the steady state concentration of the cellular oxidants at non-toxic levels [46]. This delicate balance between oxidant generation and metabolism may be disrupted by various xenobiotics including the viral proteins. This imbalance between the oxidant (e.g. ROS, SO) production and the antioxidant cellular defences cause the cell death. As expected, the oxidative stress could be observed in the later phases of BAdV-3 infection, which may be the primary factor leading to the death of the infected cells. A number of viruses including human adenovirus 5 (HAdV-5) cause oxidative stress in the cells [47-49] which has been associated with the release of progeny virus [50].

Stimulus for the mitochondria to perform beyond their usual capacity comes from various factors including stress caused to the cell by ROS. It is known that the oxidative stress causes an increase in mt DNA copy number and stimulates the nucleus to synthesize the proteins required for the mitochondrial biogenesis [51]. This scenario plays two roles in the affected cells. During initial stages (6–12 hpi) of BAdV-3 infection, when the cells are relatively healthy, they have higher antioxidant capacity and good quality of mitochondrial DNA. So, the mild oxidative stress during this phase of infection may be causing an increase in the mitochondrial DNA leading to the proliferation of mitochondria with healthy cristae. This increases the total surface area available for ATP synthesis (cristae), which in turn compensates for the increased energy supply of the cell under given conditions. Such activity has also been loosely implicated to prevent the apoptosis in human cytomegalovirus (HCMV) infected cells [52].

These mitochondria will be able to cause increase in ATP synthesis, supply energy and participate in anti-apoptotic activities. Moreover, not all the cristae are damaged during the initial phases of infection and the remaining cristae may be producing ATP at increased levels along with healthy mitochondria produced as a result of biogenesis, which explains the increase in ATP production when some of the crisate are observed to be damaged. At 18 hpi, when the oxidative stress has started to rise but it is still below threshold to produce faulty mt DNA and thus faulty mitochondria. At this time it is possible that there are some proportion of faulty mitochondria generating ROS. When ROS threshold is crossed, the cell falls into ROS loop inflicting further damage to the mitochondria (as seen in 24 h) and dies releasing the progeny virus.

Complete loss of the internal architecture of mitochondria at 24 hpi indicated that the organelle was one of the main targets of the BAdV-3 during infection. The functional analysis revealed that the functions of the mitochondria were not compromised till late during the infection. This is consistent with the fact that the completion of virus cycle requires supply of ATP. Interestingly, at late times post infection, the mitochondria were located near the protein factories (possibly virus factories). Taken together, these results suggested that the mitochondria are active till late times post infection, when they lose the internal architecture consistent with providing energy required for the release of the virus. These observations allow us to speculate that virus takes the vital mitochondrial processes under its control early during the infection and abandons the organelle once the life cycle is over.

In conclusion, our study demonstrated that there is a delicate balance between the cellular functions, the mitochondrial physiology and BAdV-3 replication. Moreover, during early stages of BAdV-3 infection, the retention of Ca^2+^ by mitochondria may prevent the loss of MMP and, decrease the SO and the ROS production by the infected cells, prolonging the cell survival for efficient production of the progeny BAdV-3 virions.

## Competing interests

The authors declare that they have no competing interests.

## Authors’ contributions

SKA was responsible for lab work, data analysis and writing of the first draft of the manuscript. JS provided expertise for EM and helped with the interpretation of the EM data. AG participated in electron microscopy studies involving preparation of samples and interpretation of the results. SKT conceived the project, designed experimental approach and writing of the manuscript. All authors read and approved the final version.
